# Exploiting the Amazing Diversity of Natural Source-Derived Polysaccharides: Modern Procedures of Isolation, Engineering, and Optimization of Antiviral Activities

**DOI:** 10.3390/polym13010136

**Published:** 2020-12-30

**Authors:** Bimalendu Ray, Martin Schütz, Shuvam Mukherjee, Subrata Jana, Sayani Ray, Manfred Marschall

**Affiliations:** 1 Department of Chemistry, The University of Burdwan, Burdwan, West Bengal 713104, India; bray@chem.buruniv.ac.in (B.R.); shuvamm82@gmail.com (S.M.); subratajanachem@gmail.com (S.J.); 2 Institute for Clinical and Molecular Virology, Friedrich-Alexander University (FAU) of Erlangen-Nürnberg, 91054 Erlangen, Germany; martin.schuetz@extern.uk-erlangen.de

**Keywords:** natural source-derived compounds, amalgamated extraction-sulfation methods, polysaccharide sulfates, chemical profiles, broad-spectrum bioactivity, specific antiviral activities, structure–activity relationships

## Abstract

Naturally occurring polysaccharide sulfates are highly diverse, owning variations in the backbone structure, linkage pattern and stereochemistry, branching diversity, sulfate content and positions of sulfate group(s). These structural characteristics bring about diverse sulfated polymers with dissimilar negative charge densities and structure–activity relationships. Herein, we start with a short discussion of techniques needed for extraction, purification, chemical sulfation, and structural characterization of polysaccharides. Processes of isolation and sulfation of plant-derived polysaccharides are challenging and usually involve two steps. In this context, we describe an integrated extraction-sulfation procedure that produces polysaccharide sulfates from natural products in one step, thereby generating additional pharmacological activities. Finally, we provide examples of the spectrum of natural source-derived polysaccharides possessing specific features of bioactivity, in particular focusing on current aspects of antiviral drug development and drug–target interaction. Thus, the review presents a detailed view on chemically engineered polysaccharides, especially sulfated derivatives, and underlines their promising biomedical perspectives.

## 1. Introduction

A variety of different macromolecules are preserved in natural ecosystems. Specifically, the world’s oceans carry significant biological diversity and a huge variety of so far unexploited marine bioproducts. On the global scale, oceans cover 70% of the earth’s surface, encompassing >90% of the known biosphere volume. It is thus an accepted concept that the immense size and range of life in the oceans point to a great potential for discovery that surpasses all other environments [1]. The main basis of biodiversity is the almost limitless abundance, and distribution of life in the ocean, also including microorganism and infectious agents, with extreme virus titers of up to 10^7^/mL in water and 10^9^/cm^3^ in sediments [2]. From the application-related and medical points of view, it is a highly attractive perspective to utilize the sea’s richness in bioactive macromolecules spanning pharmaceuticals, nutraceuticals, adhesives, biocatalysts/enzymes, and source-materials for cosmetics. Despite the greater numbers of documented species and established bioactive compounds for land biota compared with marine biota [3], the oceans nonetheless support substantially higher diversity at a higher taxonomic level, i.e., taxonomic distinctness, and thus may well eventually yield more species and a greater pool of bioactive compounds than all other environments combined [1]. Considering that many current drugs are still derived from terrestrial sources, a substantial number of drugs, drug candidates and other metabolites from marine organisms have been identified in recent years. About 30,000 compounds of marine origin are known and, since 2008, more than 1000 compounds are newly discovered each year, which are often characterized by structural novelty, complexity, and diversity [4,5]. Life in the marine environment has evolved on the basis of special conditions, which necessitate appropriate mechanisms of adaption of organisms to growing in the ocean that may be fundamentally different from those in land-based organisms. One important adaption mechanism is the production of biologically active secondary metabolites. Functions of such metabolites are diverse and may span intra- and interspecific signaling, deterrence of herbivores and predators, suppression of competing neighbors and the inhibition of bacterial or fungal invasion. These bioactive compounds thus possess an enormous potential to become drugs, medicinal products, experimental tools, or food supplements [6].

For decades, specific focus has been given to the generation of bioactive substances isolated from plant organisms, as derived from both, marine or land biota. The variety of active plant-derived products is extraordinary and covers the sources of huge numbers of algae, seaweed extracts, plant-based food, herbs, traditional medicines and many more. Plant products often have the advantage to be available at abundant quantities and low cost, some of these even received from manufacturing waste such as rice bran [7,8]. Specifically, herbal medicines and other natural product-derived phytochemicals as well reported to be potential agents against viruses of various types, also including coronaviruses as current investigations illustrated that were associated with the COVID-19 crisis [9]. In this regard, particularly the sulfated derivatives of naturally occurring polysaccharides exhibit a number of biological activities and these are highly dependent on structural compound characteristics [10,11,12,13,14,15]. Intrinsically, the composition, structure, and biological properties of polysaccharide sulfates are of deep interest for both, the basic molecular studies and the development of new products for various applications and industrial production. The manifestation of a sulfate functionality on the polysaccharide provides several chemically significant prerequisites and production-specific consequences. Firstly, sulfate groups carry a negative charge throughout a broad pH range leading to an electrostatical binding to positively charged biomolecules [16,17]. Additionally, this functionality is able to coordinate water molecules that increase and maintain tissue hydration [18,19]. Moreover, multiple sulfates on a single polysaccharide uphold an exposed extended solution conformation structure to minimize electrostatic repulsion between the negative charges [19]. Furthermore, contrary to carboxylated species, polysaccharide sulfates are able to maintain their low pH unchanged over a wide range of conditions Given the various biological activities of polysaccharide sulfates recently described by studies of our groups and other investigators, extensive research efforts are in progress to generate and translationally exploit this rich potential [19,20,21]. However, owing to the presence of a large number of stereogenic centers in these polysaccharide species, the frequent occurrence of hydroxyls with alike reactivities, and the necessity to conserve the position and configuration of glycosidic linkages, renders the synthesis of polysaccharide sulfates a challenging aspect. For this reason, from a chemical synthesis perspective, polymeric sulfated structures are generally obtained by the in vitro sulfation process of polysaccharides previously isolated from natural products, i.e., generally using a two-step process. In our analyses, however, a recently developed cost-effective one-step process revealed the ability to produce huge numbers of directly, isolation-associated sulfated derivatives of polysaccharides and, thereby inducing potential bioactive properties such as, especially, a strong level of antiviral activity in the final product [7]. These newly synthesized molecules are not only helpful in developing advances knowledge of structure–activity relationship (SAR), but also to contribute to broaden the attractive arsenal of antiviral candidate compounds. This review focuses on the chemical and biotechnological utilization of the amazing diversity of natural source-derived macromolecules, in particular polysaccharides. Specifically, the modern procedures of isolation, targeted engineering, and optimization of bioactivity of such candidate polysaccharides is discussed in the light of near-future developmental processes.

## 2. Chemical Profile of Bioactive Sulfated Polysaccharides

**Figure 1:**
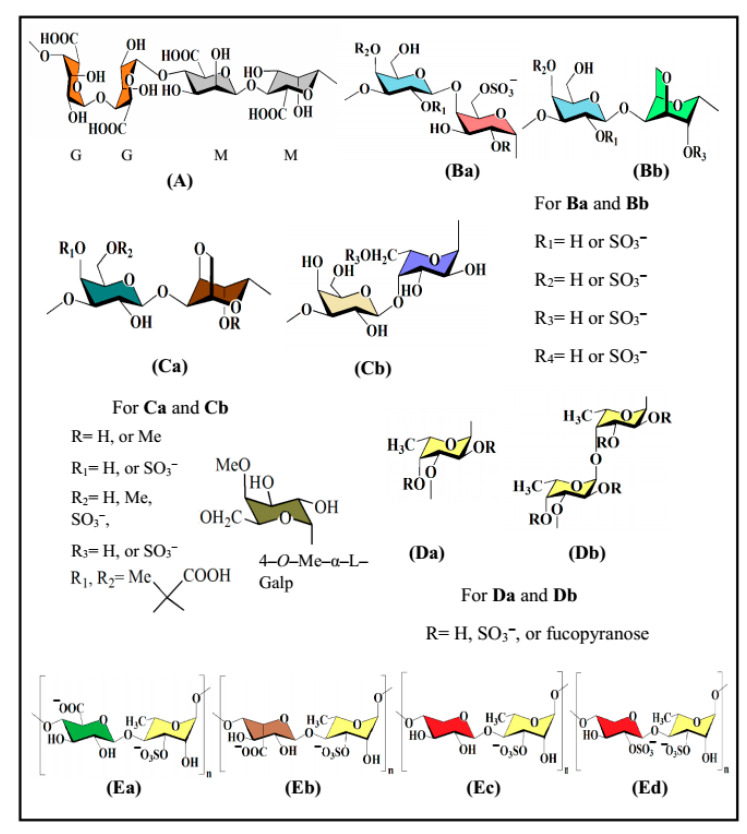
Structures of seaweed-derived polysaccharides possessing in vitro antiviral activity: alginic acid (**A**), carrageenan (**Ba**,**Bb**), agar (**Ca**,**Cb**), fucoidan (**Da**,**Db**) and ulvan (**Ea**‒**Ed**).

The biological activities, in particular antiviral activity, of naturally occurring polysaccharide sulfates are greatly reliant on the saccharide composition, glycosidic linkage pattern, backbone structure, molecular mass, and, importantly, the amount of sulfation and position of sulfate groups. Particularly, wide-spectrum activities may originate from discreet polysaccharide–protein interactions (e.g., sulfated glucans with antiviral activity) [7,11], as well as from the coordination and storage of water (e.g., lubricity). These sulfated derivatives are most abundant in and almost limited to seaweed and mammals [22,23]. Selected representatives of seaweed-derived polysaccharides possessing in vitro antiviral activities are fucoidan, carrageenan, agar, alginic acid and ulvan, and their structural features are highlighted below (Figure 1). Additional seaweed-derived polysaccharides such as xylan [24], xylomannan [25,26,27] and xyloglucan [28,29] have been described, some of which likewise possess antiviral activity, whereas other seaweed-derived polysaccharides are lacking antiviral activity.

## 3. Classical and Modern Extraction Techniques

**Figure 2:**
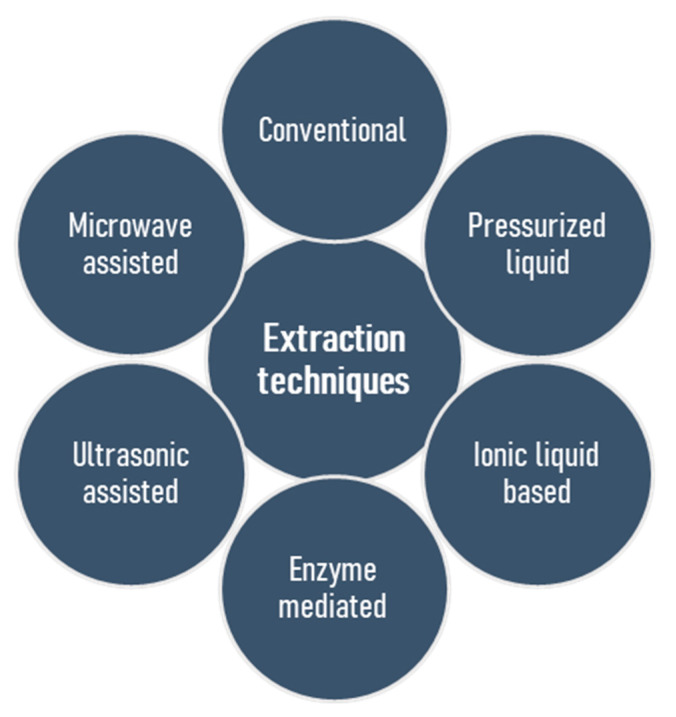
Conventional and modern procedures for the extraction of sulfated polysaccharides from marine algae.

The basic challenge, and in many cases an initial problem in the structural elucidation of naturally occurring polysaccharides, is the isolation of these substances in their natural form. Several techniques exist, but none of these appears ideal for all applications, and the establishment of a generic extraction procedure proved to be a challenging goal [30]. Moreover, seasonal variations, conditions and places of harvest are the parameters that should be taken into account [31,32]. Most extraction procedures have several features in common but are distinct in other parts, leading to individual benefits or pitfalls. Conventionally, the first stage involves solubilisation of the polysaccharides in an aqueous solvent, but precaution should be taken to ascertain that the process does not alter the polysaccharides’ structures by acid- or and alkali-catalyzed hydrolysis as well as alkali-induced β-elimination reaction. Regarding sulfated polysaccharides, the duration of extraction does not have a considerable influence on efficiency, however, long extraction time may lead to desulfation, so that a short incubation timeframe (1–3 h) is desirable [33]. Selective extraction of polysaccharides is possible by conventional methods. For example, the extraction of fucoidan using HCl or CaCl_2_ in water leads to the precipitation of alginic acid or alginates, and this strategy was found to be very useful [33,34,35]. The removal of low molecular weight compounds, exclusion of starch and protein, and then isolation of the polysaccharides by precipitation, or drying are the succeeding steps. To date, sulfated polysaccharides, which generally exert an evidently high potential of bioactivities, have most frequently been extracted from seaweed biomass by conventional water extraction methods [24,36,37,38], but a long extraction time, poor yield, low selectivity and a lack of automation are the associated drawbacks [39,40]. Heparin is the oldest clinically used anticoagulant isolated from porcine intestine and bovine lung [41]. It has a complex structure comprising of repeating disaccharide units encompassing of uronic acid residues (l-iduronic (IdoA) or d-glucuronic acid (GlcA)) and *N*-acetyl-d-glucosamine [42,43]. On the other hand, low cost levels and nontoxic water-based solvents represent the advantages of such extraction methods. The isolation of numerous bioactive polysaccharides from natural sources contributed to the improvement of classical methods and the development of modern technologies [14,39,44], which include microwave assisted extraction [45,46,47,48,49,50,51,52], pressurized liquid assisted extraction [53,54,55,56,57], ultrasonic assisted extraction [58,59,60,61,62,63,64,65], ionic liquid based extraction [66,67,68,69,70], and enzyme mediated extraction [71,72,73,74,75,76,77,78] (Figure 2). The goals of these isolation methods are to accomplish a high yield of polymer extraction, to reduce extraction time, to reduce expenditure of energy, to explore the recycling processes, to minimize structural change and to augment the scale of diversity. Table 1 highlights the pros and cons of these extraction techniques.

Modern methods provide a number of advantages over conventional procedures, including the lower volumes of required solvents, so that the term ‘green techniques’ has been introduced [14]. Notably, microwave assisted extraction is characterized by its high extraction efficiency, short extraction time and low energy consumption in an efficient process. Similarly, the enzyme-aided extraction is another of the most efficient techniques for the isolation of sulfated polysaccharides isolated from the mucus of freshwater fish Misgurnus anguillicudatus [79], fucans [34] or ulvans [80], due to the high efficiency of isolation yields when applying mild reaction conditions. Indeed, the enzyme-mediated extraction is able to convert a formerly untapped, renewable algal biomass into a valuable, ecologically sustainable resource [80].

## 4. Purification

**Figure 3:**
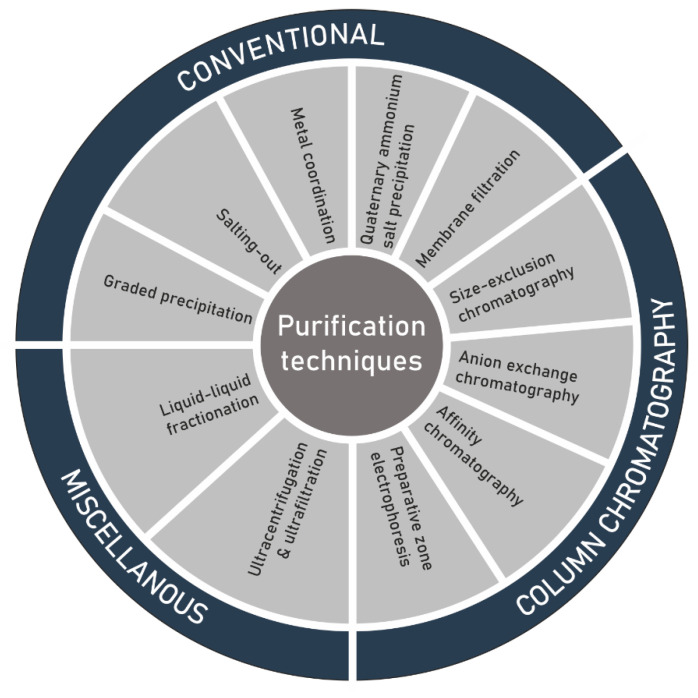
Techniques for the purification of polysaccharide sulfates extracted from seaweed.

Another challenge in the structure elucidation of polysaccharides, subsequent to extraction, is the purification of the extracted raw material (Figure 3). As a result of microheterogeneity, the definition of purity of the isolated polysaccharides is not as precise as initially thought. The polymer can connect itself to several non-carbohydrate molecules either by physical forces or through chemical bonding. An additional difficulty can be given by a limited solubility in most of the organic solvents. In most cases, purification is achieved by solvent extraction, dialysis and repetitive precipitation of the polymer from aqueous solution by using ethanol or acetone [81], antisolvent [82], or alternatively by complexation with polyvalent cations like copper/calcium, or quaternary ammonium salts such as cetylpyridinium chloride/cetyltrimethylammonium bromide [83,84,85]. Membrane filtration [86], liquid liquid fractionation, ultrafiltration and ultracentrifugation have also been used [47,87,88]. Liquid chromatographic processes such size-exclusion chromatography (SEC) and as anion-exchange chromatography (AEC) have extensively been applied for the purification before determining the structural features, physicochemical properties and biological activities of polysaccharide sulfates. AEC is mostly useful to eliminate unwarranted proteins and neutral polymers [47,89,90,91,92]. Weak anion-exchangers, such as diethylaminoethyl (DEAE), tertiary amine-functionalized media [93,94] or strong anion-exchangers including quaternary (Q) amine-functionalized media [95], have been successfully utilized to purify polysaccharide sulfates. SEC is extensively employed for the measurement of total molecular masses and molecular mass distributions [96,97,98,99,100]. Moreover, this analytical technique has been effectively employed in many examples for the elucidation of fine-structures of oligosaccharides and polysaccharides [94] and to produce size classes for the determination of structure–activity relationship [101,102,103].

## 5. Techniques for Structural Characterization of Polysaccharides

**Figure 4:**
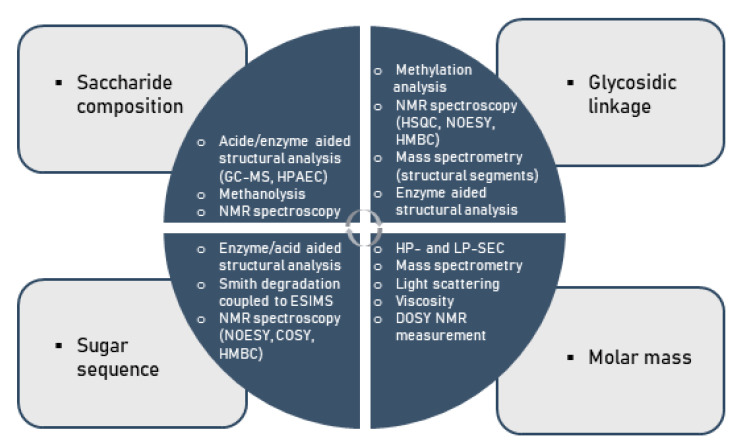
Methods for the chemical characterization of polysaccharides.

Considering the multifold modes of monosaccharide-based polysaccharide and particularly glycan linkages, variations in saccharide compositions and sequences, linkage regio- and stereo-chemistry as well as branching diversity produces an enormous structural diversity. As a consequence of this complexity, the comprehensive assignment of covalent structures of naturally occurring polysaccharides is a major challenge to the organic chemist [104,105,106,107,108]. Indeed, no method available for structural analysis will offer sufficient data to allow the structure of a polysaccharide to be defined in the above-mentioned terms. This information can only be obtained using a number of techniques, particularly in conjunction with one another, as illustrated below (Figure 4).

In specific terms under standard conditions, the analysis of the structure of a given polysaccharide begins with its hydrolysis into constituent monosaccharides. Subsequently, the liberated monosaccharides are then analyzed by approved methods such as gas chromatography–mass spectroscopy (GC-MS) or high-performance liquid chromatography (HPLC) [96,109,110,111]. The glycosidic linkages within polysaccharides are normally determined by methylation analysis with the classical Hakomori method or a modification thereof [112,113,114,115,116,117,118]. It involves the following steps: firstly, free hydroxyl groups of the polysaccharide constituents are fully substituted with methoxyl groups; secondly, the permethylated polysaccharide thereby formed is depolymerized by acid hydrolysis to a mixture of constituents including partially methylated monosaccharides; finally, each component of the mixture is then converted into partially methylated alditol acetates (PMAAs) as usually examined by GC-MS.

The glycosyl linkage patterns can be deduced from the MS fragmentation patterns of PMAAs and from the retention times [116,119]. The ring scaffold of monosaccharide units (i.e., furanose or pyranose), which occupies terminal positions in such polymers, is established by the *O*-methyl substitution patterns arising from methylation analysis. Once the glycosidic linkages of the individual monosaccharide residues within the polysaccharides are known, the sequence in which they are linked to each other has to be determined. Fragmentation of polysaccharide molecules, either by mild acid hydrolysis or enzyme hydrolyses into oligosaccharides, and the characterization of the purified fragments, is often employed to determine the sequence of monosaccharide residues [115,120]. The oligosaccharides generated are fractionated and purified by size exclusion chromatography [7,8,25,121,122,123,124], HPAE-PAD chromatography, or other techniques. Precise structures of these purified oligosaccharides may be determined by sugar compositional analysis, glycosidic linkage composition, periodate oxidation, NMR, MALDI-TOF-MS or other methods [125,126,127].

Recently, a method based on ESI-MS analysis of peracetylated oligomeric portions, as derived from polysaccharides by Smith degradation or by enzyme hydrolysis followed by acetylation, has been described as an expedient tool for providing critical structural information on a spectrum of oligosaccharides [37,128,129,130,131]. Moreover, nuclear magnetic resonance (NMR) spectroscopy is an effective, non-invasive method in general studies on biopolymers, and NMR is a particularly valuable technique in the determination of molecular structures of polysaccharides [132,133,134,135,136,137,138,139,140]. This spectroscopic technique provides information not only on the saccharide composition, glycosidic linkage patterns and sugar sequences, but also on its molecular mass. As a specific aspect of polysaccharides comprising pronounced levels of bioactivity, the presence of sulfate groups in polysaccharides creates additional challenges in deducing their structure, since the position of sulfation and the degree of sulfation (DS) needs be additionally determined. Traditional methods for the determination of sulfate contents in polysaccharides include the HPLC analysis of sulphates [94], turbidometric barium chloride method [7,8,25,122,124] and IR spectrometry [7,8,25,121,122,123,124]. The position of sulfate groups can be determined conventionally by comparison of methylation analysis results obtained for the polysaccharide sulfates with those for its desulfated derivatives [7,8,121,123,124,130]. Infrared spectroscopy (IR) spectroscopy, ^1^H and ^13^C nuclear magnetic resonance (NMR) spectroscopy may provide additional information on the position of sulfate groups [8,121,123,137,138,139,140].

## 6. Sulfate-Specific Modification of Polysaccharides

**Figure 5:**
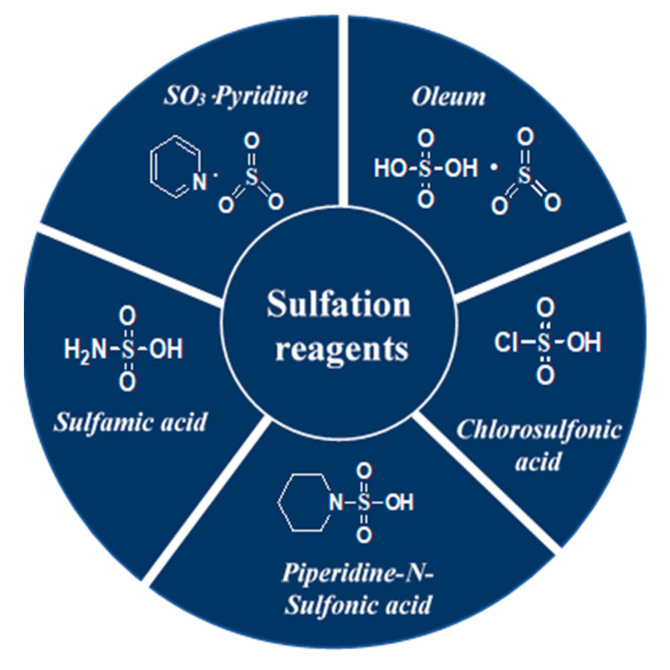
Structures of a selection of sulfation reagents.

Several techniques useful for the enrichment of sulfated polysaccharides have been applied so far. The feeble reactivity of the hydroxyl functionality, the solubilisation of both the polysaccharide and its sulfated derivatives in the same organic solvent, the preservation of the glycosidic linkage, the regioselectivity, and the isolation of the products, however, are challenges associated with polysaccharide sulfation. Significant research endeavors are directed towards sulfation of polysaccharides [141,142] and both the advantages and limitations have been recognized dependent on the characteristics of the source materials [19]. A selection of typical reagents namely, oleum [7,8,123,143], chlorosulfonic acid [141,144,145,146,147,148], sulfamic acid [149,150,151,152,153,154], piperidine-*N*-sulfonic acid [155,156,157] and SO_3_-pyridine [25,121,122,158,159,160,161,162] used in these sulfation processes is highlighted in Figure 5. The benefits and drawbacks of the sulfation modification techniques are presented by Table 2.

## 7. Targeted Engineering

While a huge number of polysaccharide types occur in living organisms, the existence of their sulfated derivatives is limited to marine algae and mammals [22,163]. Polysaccharide sulfates from plant sources may thus be produced by targeted engineering using a two-step procedure: an initial isolation of polysaccharides from the plant source materials, followed by a sulfation reaction on the isolated polymer. Recently, we developed a single-step approach for the direct production of sulfated glucans from rice bran, i.e., carrying out an amalgamated extraction-sulfation procedure using an oleum-DMF reagent [8]. A benefit of this process is conferred by the usage of DMF−fuming H_2_SO_4_ as a ’double agent’, which extracts the polysaccharide effctively from the source material and at the same time promotes the warranted modification with sulfate groups. Regarding the methodology, DMF being a polar aprotic solvent is competent in extracting the polysaccharides, which represent polar constituents comprising of hydroxyl and other polar groups. Hereby, fuming H_2_SO_4_ is expected to promote the solubilization process by disrupting the hydrogen and ionic bonds, mainly presented in the cell-wall constituents of plant material. This procedure has been validated, on the one hand, by its cost-effectiveness and, on the other hand, by generating a variety of sulfated polysaccharides with diverse building blocks from natural sources. Importantly, the obtained polymers may possess significant characteristics of bioactivity, especially antiviral activity, since the respective studies indicated that the functionalization with sulfate groups strongly induced antiviral activity [7,8]. Thus, the strategy allows for the production of bioactive macromolecules via chemical diversification and functionalization of natural source materials, typically composed of compounds rich in number and diverse in scaffolds [164,165]. Notably, such a facile procedure may be ultimately standardized and made applicable for larger-scale processes. Thus, specifically our experiences with a one-step sulfation procedure may stimulate further research projects to apply this experimental approach in related studies, likewise generating biologically active compounds, and possibly aiming at a pharmaceutical development of natural source-derived drugs. This integrated extraction-sulfation technique is efficient and can provide plentiful material for biological assays and for comparative studies to naturally occurring polysaccharide sulfates.

## 8. Current Focus on Antiviral Activity: The Spectrum of Natural Source-Derived Bioactive Polysaccharides

To date, a huge variety of natural sources, such as marine flora and fauna, bacteria, fungi and higher plants have been utilized for the screening of suitable natural substances for medical or biochemical use. In particular, marine algae are considered a promising source for bioactive compounds, as they are increasingly used and studied for medical purposes [6,166]. The antiviral potential of polysaccharides was first discovered by Ginsberg et al. in 1947, where they showed an inhibition against mumps [167]. Eleven years later in 1958, Gerber and colleagues could demonstrate an inhibition of mumps and hepatitis virus B by marine algae-derived polysaccharides [168]. Since that finding, a number of carbohydrate compounds from marine algae, cyanobacteria, and animal sources were described showing potent inhibitory effects against several human and animal viruses [169,170,171,172,173,174]. A selection of promising natural substances will be discussed in the following section. Fucan sulfates from marine brown algae are normally structured in a complex and heterogenous manner. However, these polysaccharides consistently contain a backbone of either α-(1→ 3)-linked or changing α-(1→ 3)- and α-(1→ 4)-linked L-fucopyranosyl residues with sulfate groups at position 4 [35,175,176,177,178]. This backbone is in many cases masked by monosaccharides (galactose, glucose, mannose, xylose or glucuronic acid), acetyl groups, and/or sulfate esters. Fucan sulfates have potent antiviral activity against herpes simplex virus type 1 (HSV-1) and herpes simplex virus type 2 (HSV-2) without effects of cytotoxicity in cell culture [124,179,180,181,182]. In contrast, polysaccharides from red seaweeds consist of linear sulfated galactans containing alternating β-(1→ 3)-d- and α-(1→4)-galactopyranosyl residues [183,184,185]. These galactans can be distinguished by the configuration of the α-linked units: polymers containing the L-type configuration are termed agaran; For D-type configuration it is carrageenan. Agaran has been shown to exert antiviral activity against HSV-1 [186], carrageenans show activity against HSV-2 and dengue virus [187]. Moreover, DL-hybrid galactan sulfate, a polymer in which α-linked units can have D- and L-configuration in the same molecules, has been shown to have strong antiviral activity against HSV-1 and dengue virus [188]. Concerning marine green algae, polysaccharides called ulvan are of high research interest for the development of new therapeutic agents. It consists mainly of sulfate, rhamnose, glucuronic acid, iduronic acid, and xylose; the major repeating disaccharide units are glucuronic acid and rhamnose 3-sulfate, and iduronic acid with rhamnose 3-sulfate [94,189,190,191,192]. As other sulfated polysaccharides, ulvan can block the adsorption of the virus and thereby block the viral entry into the cell. Ulvan has been demonstrated to have activity against the Japanese encephalitis virus and the influenza virus A (IAV) and HSV-1 [123,193,194]. Seen apart from the specific topic of sulfated polysaccharides, another promising biopolymer, called alginate, can either be derived from brown algae or several bacteria [195,196]. Alginate consists of β-(1→ 4-d-mannuronic acid (M) and α-(1→ 4)-l-guluronic acid (G) residues, which can either be arranged in a homogenous (poly-G, poly-M) or heterogeneous (MG) manner [197,198,199]. The alginate-derived drug 911 exhibits activity against human immunodeficiency virus type 1 (HIV-1) by inhibiting the viral reverse transcriptase [200], against hepatitis B virus by inhibiting the viral polymerase [201] and HSV-1 [36]. Alginate polymers also showed antiviral activity against other viruses, such as HSV-1 and HSV-2 or the human papillomavirus [202,203,204]. Specifically, Sinha et al. (2010) reported that the chemically sulfated derivatives of a guluronic acid-rich alginate derived from Sargassum tenerrimum showed activity against herpes simplex virus type 1 (HSV-1) by mimicking the active domain of the entry receptor [205]. Sulfated polysaccharides with antiviral activity are found rarely in higher plants. Therefore, original natural substances can be chemically engineered via a two-step procedure: the initial isolation of the polysaccharide of interest followed by chemical sulfation. These chemically modified natural substances offer interesting pharmacological alternatives for antiviral drug development. Importantly, our group developed a novel single-step strategy for the direct production of sulfated glucans from rice bran [8]. The high antiviral potential of sulfated glucans could be repeatedly underlined by a pronounced activity against the human cytomegalovirus (HCMV), HSV-1, HSV-2, respiratory syncytial virus, IAV and HIV-1 [7,122,206,207]. Also other natural substances are target of chemical modifications, such as arabinogalactan, which can serve as a carrier of therapeutic agents [208] or xylomannan, which either in its natural form or artificially over-sulfated form exerts strong antiviral activity against HSV-1 [25,27,209].

## 9. Structure–Activity Relationship of Sulfated Glucans with Antiviral Activity

A large number of sulfated polysaccharides possessing antiviral activities has been described in the last decades. However, substantial structural differences between the various bioactive compounds and limited data made it difficult to establish a convincing structure–activity relationship (SAR). Nonetheless, based on recent findings, several common structural motifs can be considered to be generally important for antiviral activity. A special focus has been directed to sulfated glucans. One major determinant of antiviral activity is (i) the molecular weight (MW) of the sulfated polysaccharide. In general, high MW often indicates strong antiviral activity. Semisynthetic glucans with MWs ranging between 1 and 500 kDa had the highest antiviral activity for the higher MW fractions, even though no increased antiviral activity was observed for fractions higher than 100 kDa [210]. This correlation can also be observed for naturally occurring polysaccharides, like agarans [186], carrageenans [211], fucans [212] or chemically sulfated polysaccharides from Azadirachta indica leaves [121]. In a similar fashion, (ii) chain length can also play a major role for a high compound activity. In such manner, the long chains of CS-E (approx. 70 kDa) blocks viral entry of HSV-1 at lower concentrations than heparin chains (approx. 12.5 kDa), even though DS of CS-E is lower (approx. 1.7 sulfates/disaccharide) than that of heparin (approx. 2.7 sulfates/disaccharide) [213]. Unfortunately, derivatives with high MW can have the disadvantage of poor tissue-penetrating capabilities and are therefore often not suitable for applications in humans [214]. To address this problem, oligosaccharides, e.g., carrageenan, can be chemically and enzymatically degraded, which improves bioavailability and biological activity significantly [215]. Another major determinant is (iii) the degree of sulfation (DS; i.e., the amount of sulfate groups per monosaccharide) [216]. The obvious correlation between intensively investigated naturally sulfated polysaccharides, such as carrageenan or fucans, and their antiviral activity despite different structures underlines the relevance of DS. This also applies for chemically sulfated glucans, where the antiviral activity also differs significantly depending on the DS, i.e., highly sulfated glucans being generally more active. Removal of the sulfate functionality from glucans drastically reduced the antiviral activity [7]. Current research suggests that this correlation is mainly due to electrostatic interactions between positively charged regions of the viral glycoprotein and cellular HS chains on the cell surface. A highly charged molecule, such as strongly sulfated saccharides, are more likely to interfere with this interaction [11]. Chemically sulfated glucans, however, showed antiviral activity even when added after virus adsoroption/infection, suggesting additional modes of action [7]. In addition, (iv) the distribution of sulfate moieties is important, since an overall barely sulfated polysaccharide still can possess certain highly charged parts in the polysaccharide backbone and, therefore, exert antiviral activity [11]. Nonetheless, recent studies with glucans showed that DS and high MW alone are not sufficient to make an effective compound, since derivatives with various MW and an identical degree of sulfation of glucose, maltose and β-cyclodextrin were not able to interfere with HCMV entry and replication [7]. In a similar direction, carrageenan with similar sulfate contents (approx. 50 mol%) derived from different sources (Callophyllis variegate, Gigartina skottsbergi, Gymnogongrus griffithsiae and Meristiella gelidium) showed different degrees of antiviral activity [187,211,217,218]. This phenomenon could be explained with the charge density, since it correlated with the antiviral IC_50_ values against HSV-1 in fractions obtained from the same marine algae [211]. Next, (v) the overall sugar composition and chemical structures have an impact on antiviral activity of chemically sulfated glucans: sulfated glucans containing no uronide and high DS of 1.7 showed higher antiviral activity compared to saccharides containing high amounts of uronic acid residues (9%, *w*/*w*) and DS of 1.2. For this reason, anionic sulfate groups seem to be important for the antiviral activity of substances, in contrast to carboxyl groups, which do not have a comparable impact [7]. To summarize, recent data reported by our group and other research clearly illustrated that antiviral properties of sulfated polysaccharides are not only a question of charge density, DS and MW, but also of the compounds’ detailed structural features, which will have to be elucidated even further in the upcoming years [7,8,11,219].

## 10. Future Perspectives: A Specific Focus on COVID-19 and Other Emerging Viral Diseases

The COVID-19 pandemic, which spanned over 2020 in a devastating graveness that is casting shadows into 2021 and the following years, requires novel strategies of rapid and strain-crossing treatment and prevention. Natural source-derived polysaccharides might provide one of such keys for the concise control of another early epidemic or pandemic development [72,220]. Notably, the SARS-CoV-2 pandemic resembled the mode of dissemination of influenza viruses in several aspects, as both viruses use sialic acids (SA). Currently, information on SARS-CoV-2 and its receptors is limited, although the human ACE-2 surface protein (uses angiotensin-converting enzyme 2) is considered as the general viral entry receptor [221]. In addition, O-acetylated SAs interact with the lectin-like spike glycoprotein (S) of SARS-CoV-2 for the initial virus attachment to the host cell. Moreover, SARS-CoV-2 hemagglutinin-esterase (HE) acts as the classical glycan-binding lectin and receptor-degrading enzyme. Most CoVs recognize 9-O-acetyl-SAs, but occasionally switched to recognizing the 4-O-acetyl-SA form during virus evolution. Type I HE is specific for the 9-O-Ac-SAs and type II HE is specific for 4-O-Ac-SAs [222]. In either case, competitive use of pharmacologically developed polysaccharide products may interfere with virus-host cell binding and might thus act as a topical therapeutical measure to prevent surface binding of SARS-CoV-2 or related CoVs particularly considering mucosal body surfaces. This concept is based on the known fact that the first attachment step of viral infection is initiated on the respiratory cell surfaces, driven by the viral S protein, thus postulated as a potential therapeutic target. In SARS-CoV-2, the ACE-2 receptor interacts with the S glycoprotein cleaved by a specific serine protease. SARS-CoV-2 infection is regulated by glycosylated SARS-CoV-2 viral particles and glycosylated ACE-2 in the lung epithelial cells. Recently, anti-CoV drugs have been approached using molecular modeling, docking and simulation methods. Computation-assisted drugs via molecular modeling and docking toward drug targets are applied as antiviral compounds against CoVs. In this manner, a marine product-derived library containing more than 14,000 compounds was recently analyzed for antiviral activity against the essential main protease of SARS-CoV-2, revealing 17 potential hit substances with inhibitory activity [223]. Intriguingly, most of these selected substances belonged to the class of phlorotannins and were derived from Sargassum spinuligerum brown algae, which are extensively used in the traditional Chinese medicine (TCM) [224]. Also other TCM formula, such as Lianhuaqingwen, which is composed of 13 different herbs, showed strong anti-SARS-CoV-2 activity in cell culture-based test systems and is therefore currently considered for clinical applications in China in comparison to or combination with alredy existing therapeuticals [225]. The applications of TCM have moved into a major focus of interest, particularly in the face of the COVID-19 pandemic and the perspectives of current investigations and near-future use [226]. Thus, natural source-derived polysaccharides are thorougly investigated at the moment, among which carrageenan is considered as one of the most promissing. The prophylactic efficacy of ioata-carrageenan for health care professionals is currently investigated in a phase III clinical trial (NCT04590365). The potent efficacy of iota-carrageenan has previously been demonstrated in clinical trials against virus-confirmed common cold (caused by rhinovirus, coronavirus or IAV), reducing both the duration of disease and the number of relapses significantly [227]. Importantly, natural substances might not exclusively be applied for antiviral treatment but may also prove to become helpful in the prevention of pulmonary fibrosis, which is frequently developed during the post-symptomatic phase succeeding a viral respiratory infection, such as cases of SARS-CoV infection in 2003. This potentially life-threatening disesase is caused by a hyperactive host response caused by epidermal growth factor receptor (EGFR) signaling [228]. Several sulfated polysaccharides such as fucoidan have the ability to inhibit the expression or the downstream pathway of EGFR, which may therefore support the prevention of consequential damages of SARS-CoV-2 infection [229,230]. These points illustrate the broad potential of natural source-derived polysaccharides. Combined, future antiviral applications might not only include direct antiviral inhibitory effects but might also target antiviral infections indirectly or follow-up diseases. On the basis of modern procedures of an optimization of bioactivity, the translational exploitation of polysaccharide products may open up many so far untapped opportunities of compound development.

## Figures and Tables

**Table 1:** Advantages and disadvantages of polysaccharide extraction techniques.

Extraction Techniques	Advantages	Disadvantages	References
Conventional water extraction	Simple and low-cost equipment Ease of operation	Limited yield and selectivity Long extraction time High energy consumption Lack of automation	[24,36,37,38]
Pressurized liquid assisted extraction	Selective solubilization by modulating operation severity Shorter extraction time	Equipment costs Degradation of thermolabile compounds Hydrolysis of polymers	[53,54,55,56,57]
Ultrasonic assisted extraction	Low cost Reduction of extraction time Low solvent consumption Reproducibility and scalability Possibility to scale up to industry requirements	Noise pollution Lack of uniformity in the vessel Uneven heating in the vessel Long-term ultrasonication possibly inducing structural changes	[58,59,60,61,62,63,64,65]
Ionic liquid extraction	High extraction efficiency ‘Green’ solvents Negligible emulsion formation Minimal viscosity Fast phase separation	High cost factor Very high corrosiveness Highly hygroscopic	[66,67,68,69,70]
Microwave assisted extraction	High extraction efficiency Short extraction time Low energy consumption	Degradation of thermolabile compounds	[45,46,47,48,49,50,51,52]
Enzymatic assisted extraction	High specificity High catalytic efficiency Mild reaction conditions Low energy consumption Food-grade enzymes	Limited enzyme recycling Enzyme costs Availability of suitable enzymes	[71,72,73,74,75,76,77,78]

**Table 2:** Benefits and drawbacks of sulfation modification techniques.

Sulfation Reagents	Benefits	Drawbacks	References
Oleum	Broad DS values ^a^ Low toxicity	Corrosive reagent, may lead to polysaccharide degradation Strongly oxidizing agent Powerful dehydrating agent	[7,8,123,143]
Chlorosulfonic acid	High DS values High yields	Reaction difficult to control Long reaction times Complicated operation Strongly oxidizing agent	[141,144,145,146,147,148]
Sulfamic acid	Mild reaction conditions Low degradation of products	Low DS values Low yields High temperatures may lead to degradation	[149,150,151,152,153,154]
Piperidine-N-sulfonic acid	Mild reagent Low degradation of products	Slow reaction	[155,156,157]
SO_3_-pyridine	Ease of handling Compatibility with polar solvents Regioselective sulfation by modulating reaction conditions Stability of reaction conditions High DS values Mild reaction conditions	High costs Oxidizing agent	[25,121,122,141,158,159,160,161,162]

^a^ DS, degree of sulfation.
